# Intra-annual density fluctuations in tree rings are proxies of air temperature across Europe

**DOI:** 10.1038/s41598-023-39610-8

**Published:** 2023-07-29

**Authors:** G. Battipaglia, J. P. Kabala, A. Pacheco-Solana, F. Niccoli, A. Bräuning, F. Campelo, K. Cufar, M. de Luis, V. De Micco, M. Klisz, M. Koprowski, I. Garcia-Gonzalez, C. Nabais, J. Vieira, P. Wrzesiński, N. Zafirov, P. Cherubini

**Affiliations:** 1 Department of Environmental, Biological and Pharmaceutical Sciences and Technologies, University of Campania ‘L. Vanvitelli’, Via Vivaldi 43, 81100 Caserta, Italy; 2 The Earth Institute, Tree-Ring Laboratory, Lamont-Doherty Earth Observatory of Columbia University, New York, 10964 USA; 3 Institute of Geography, Friedirich-Alexander University Erlangen-Nürnberg, Wetterkreuz 15, 91058, 91054 Erlangen, Bavaria Germany; 4 Department of Life Sciences, Centre for Functional Ecology, University of Coimbra, Calçada Martim de Freitas, 3000-456 Coimbra, Portugal; 5 Department of Wood Science and Technology, Biotechnical Faculty, University of Ljubljana, Jamnikarjeva Ulica 101, 1000 Ljubljana, Slovenia; 6 Department of Geography and Regional Planning. Environmental Sciences Institute (IUCA), University of Zaragoza, Calle Pedro Cerbuna 12, 50009 Zaragoza, Spain; 7 Department of Agricultural Sciences, University of Naples Federico II, Via Università 100, 80055 Portici, Italy; 8 Dendrolab IBL, Department of Silviculture and Forest Tree Genetics, Forest Research Institute, Braci Leśnej 3, Sękocin Stary, 05-090 Raszyn, Poland; 9 Department of Ecology and Biogeography, Faculty of Biological and Veterinary Sciences, Nicolaus Copernicus University, Ul. Lwowska 1, 87-100 Torun, Poland; 10 BIOAPLIC, Departamento de Botánica, EPSE, Universidade de Santiago de Compostela, Campus Terra, 27002 Lugo, Spain; 11 ForestWISE, Collaborative Laboratory for Integrated Forest and Fire Management, Quinta de Prados, 5001-801 Vila Real, Portugal; 12 Department of Plant Pathology and Chemistry, University of Forestry, Sofia, Bulgaria; 13 Swiss Federal Institute for Forest, Snow and Landscape Research WSL, Zürcherstrasse 111, 8903 Birmensdorf, Switzerland; 14 Department of Forest and Conservation Sciences, Faculty of Forestry, University of British Columbia, 2004-2424 Main Mall, Vancouver, BC V6T 1Z4 Canada

**Keywords:** Climate-change ecology, Forest ecology, Ecology, Climate sciences, Palaeoclimate

## Abstract

Intra-Annual Density Fluctuations (IADFs) are an important wood functional trait that determine trees’ ability to adapt to climatic changes. Here, we use a large tree-ring database of 11 species from 89 sites across eight European countries, covering a climatic gradient from the Mediterranean to northern Europe, to analyze how climate variations drive IADF formation. We found that IADF occurrence increases nonlinearly with ring width in both gymnosperms and angiosperms and decreases with altitude and age. Recently recorded higher mean annual temperatures facilitate the formation of IADFs in almost all the studied species. Precipitation plays a significant role in inducing IADFs in species that exhibit drought tolerance capability, and a growth pattern known as bimodal growth. Our findings suggest that species with bimodal growth patterns growing in western and southern Europe will form IADFs more frequently, as an adaptation to increasing temperatures and droughts.

## Introduction

Tree rings are comprised of the wood formed over the span of a growing season. Trees growing in temperate climates produce a light band of less dense earlywood during the spring and a dark band of denser latewood during the summer. A single tree ring corresponds to a single calendar year^1^. Annual weather patterns drive the onset and cessation of cambial activity and tree-ring growth rates. Such regularly formed tree rings enabled the development of the field of dendrochronology^1^. However, regular annual tree rings are not formed in many ecosystems^2^, such as in humid tropical areas where the cambium remains active throughout the year^3,4^,even if much progress has been achieved in recent years to identify tropical tree species that form annual rings^5^. There are also climatic zones where weather regimes may halt cambial activity more than once a year, making it difficult to assign specific dates to individual rings^6^. These types of rings have been referred to as false rings, double rings, growth zones, intra-annual rings, or rings with intra-annual density fluctuations (IADFs), and are characterized by several successive earlywood- and latewood-like bands^6–11^.

IADFs are commonly formed in trees growing in the Mediterranean region, and were initially thought to constrain the application of dendrochronology in regions characterized by Mediterranean climates^12–15^. However, IADFs have attracted increasing attention over the last two decades because they reflect variations in climatic conditions during the growing season, and can therefore be used as proxies of past environmental conditions with intra-annual resolution^11,16–18^. To date, studies focusing on IADFs formation have mainly been concentrated within the western Mediterranean basin, and have focused primarily on the genus *Pinus* and a few other gymnosperms^11,16,18–22^. A smaller number of studies have focused on angiosperms, such as *Arbutus unedo*, *Erica arborea* and *Quercus ilex*^2,23–28^. The frequency of IADFs seems to be related to age and tree-ring width effect^8,11,16,17^ and to intra-annual variability in climatic conditions or in soil water availability, especially when a dry summer period is followed by mild and wet conditions in autumn^16,29^. However, most studies have considered only a few sites or focused on a single species along a transect. A comprehensive analysis using a larger database that includes a variety of species growing under different site conditions is still missing. Previously published studies using dataset on IADFs showed only three pine species growing on sites across Italy and Iberian Peninsula^29^, or used only part of the present dataset in a qualitative way and without exploring relationships between climate and IADFs formation^17^.

Understanding how climate variations drive the formation of IADFs at continental scales will help forest managers identify species that are well-adapted (in terms of resilience and resistance) to the expected increase in heatwaves and drought events. In this study, we collected data on IADFs frequency at the continental scale over Europe with the goal of identifying the climatic or environmental factors that trigger IADFs formation. Our main hypothesis is that extreme climatic conditions in terms of low precipitation or high temperatures influence wood formation at the intra-annual level. Such events trigger an increase in IADFs, with trees in drier southern areas being more prone to IADFs formation as compared to trees growing in more humid northern regions.

## Results

### Species composition and geographical distribution

About 20% of all examined tree rings (121,556) from a total of 4,275 tree-ring series contain IADFs in the common period 1979–2000.

**Figure 1:**
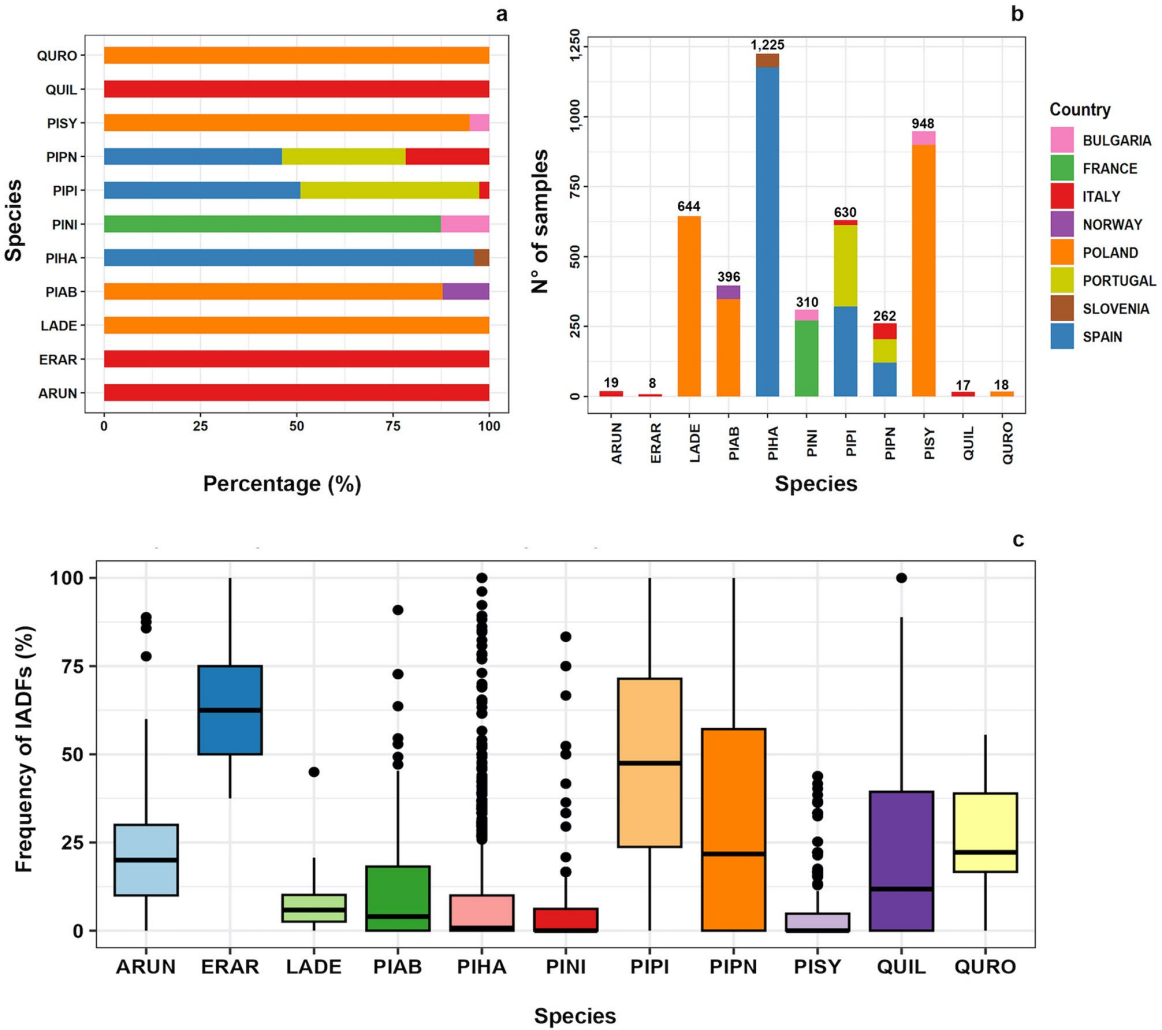
Distribution of study sites, per country and species. (**a**) Percentage of each species per country. (**b**) Absolute number of sampled trees of each species per each country. (**c**) Boxplot showing the distribution of the frequency of IADFs recorded in the different species. ARUN = *A. unedo*; ERAR = *E. arborea*; LADE = *L. decidua*; PIAB = *P. abies*; PIHA = *P. halepensis*; PINI = *Pinus nigra*; PIPI = *P. pinaster*; PIPN = *P. pinea*; PISY = *P. sylvestris*; QUIL = *Q. ilex*; QURO = *Q. robur.*

Some species are studied at multiple sites (Fig. 1a), whereas others are studied at a single site (e.g., *E. arborea*) and are therefore less well-represented in this study despite of their broad distribution range. There is also a considerable difference in the absolute number of analyzed trees (Fig. 1b) with *Pinus halepensis* sampled in high numbers across two countries. The highest mean percentages are found in *Pinus pinaster* (47%) and *Pinus pinea* (29%) for gymnosperms, and in *E. arborea* (64%), *A. unedo,* and *Quercus robur* (around 25%) for angiosperms (Fig. 1c).

**Figure 2:**
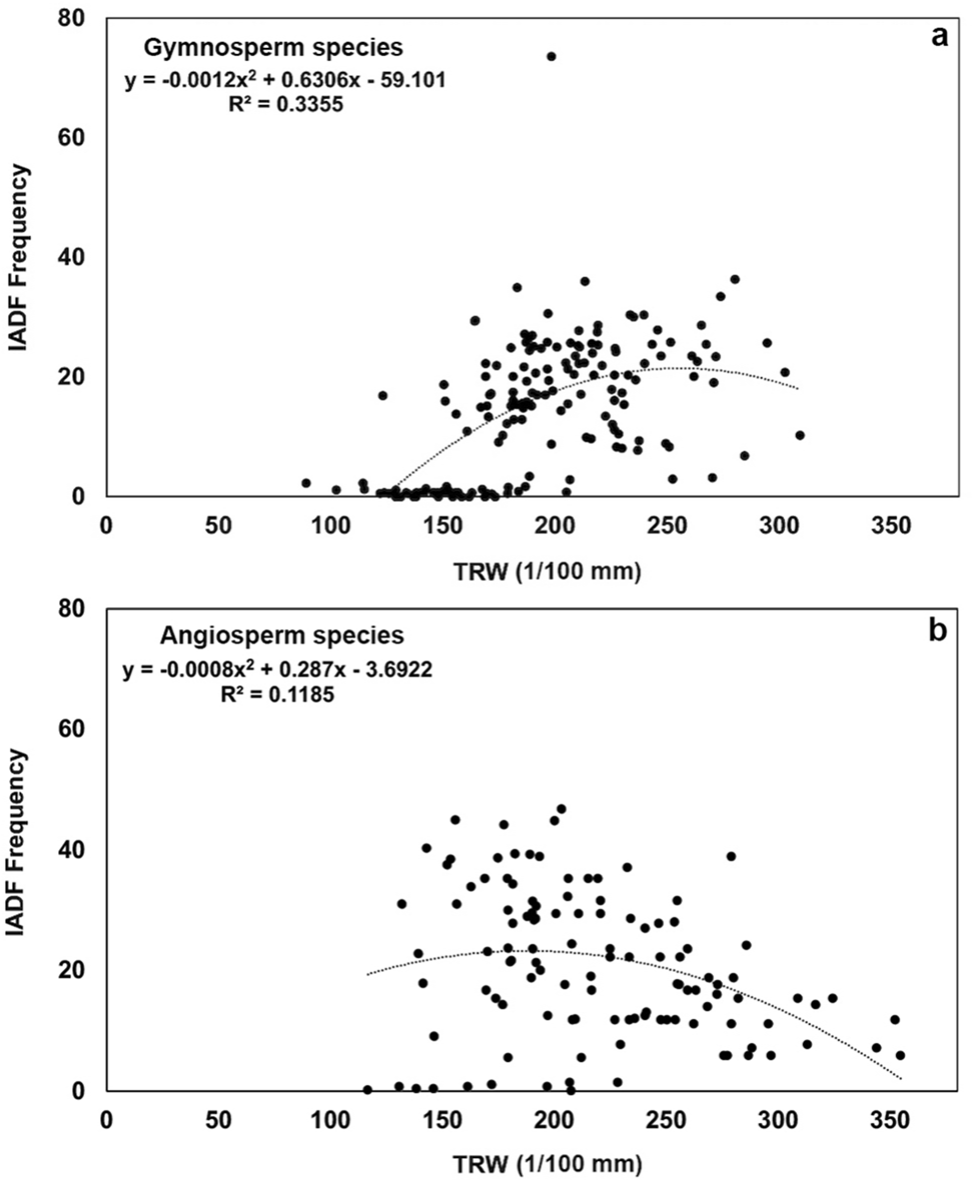
Variation in the frequency of IADFs as a function of tree-ring width (TRW) in gymnosperm and angiosperm tree species. (**a**) IADFs frequency as a function of tree-ring width in gymnosperm species (y = − 0.0012x^2^ + 0.6306x − 59.101; *p* < 0.05). (**b**) IADF frequency as a function of tree-ring width in angiosperm species (y = − 0.0008x^2^ + 0.287x + 0.1009; *p* < 0.05).

The occurrence of IADFs in relation to tree-ring width is bell-shaped for all species, with a higher frequency of IADFs in wider rings as compared to narrower rings (Fig. 2a, b). IADFs occur most frequently in gymnosperms when tree-ring widths range between 1 and 2 mm, and in angiosperms when tree-ring widths range between 2 and 3 mm.

**Figure 3:**
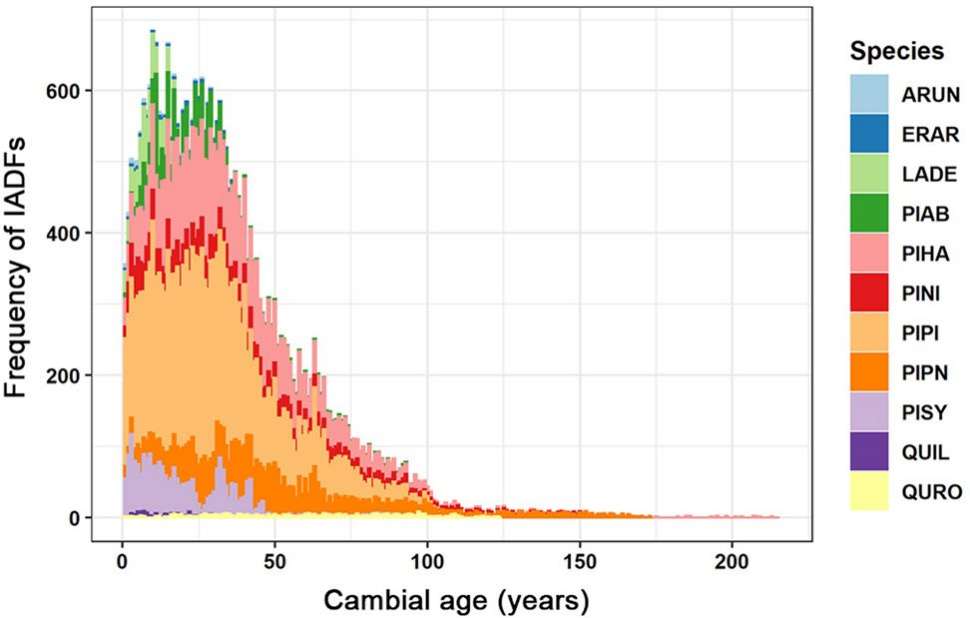
Cumulative IADF frequency for all species in the network in relation to cambial age. IADFs frequency for all species in relation to cambial age. Species abbreviations as in Fig. 1.

The frequency of IADFs in all species exhibits an age trend (Fig. 3), with IADFs forming more frequently while trees are young (around 30 years).

**Figure 4:**
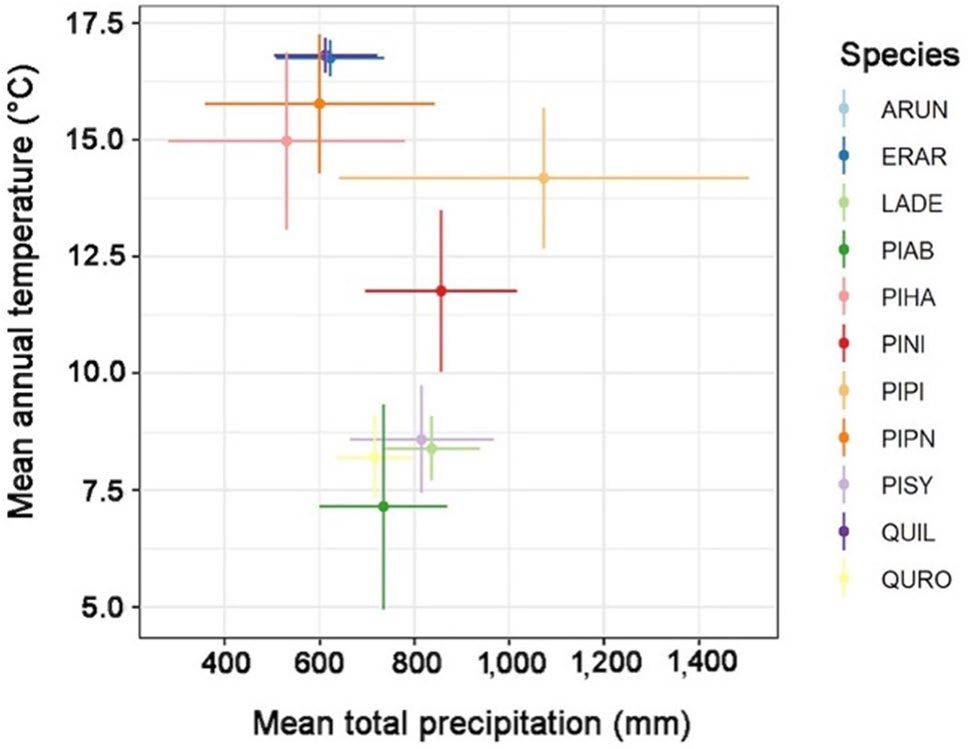
Species distribution in relation to mean annual temperature and mean total precipitation. Species distribution. The points represent the average mean annual temperature and the average total annual precipitation. Error bars identify ± 1 standard deviation from the average, providing an indication of the broadness of the considered climatic conditions. Abbreviations as in Fig. 1.

The climatic conditions of the sites at which each species was sampled are reported in Fig. 4. Mediterranean species, such as *A. unedo*, *E. arborea*, *Q. ilex*, *P. pinea* and *P. halepensis* occur under climate conditions with high temperatures and limited mean total annual precipitation, while *P. pinaster* is found across a broad range of climate conditions. In the lower part of the graph with high amount of annual precipitation and lowest temperature, boreal and temperate species like *Picea abies*, *Larix decidua*, *Q. robur* and *Pinus sylvestris* occur.

**Figure 5:**
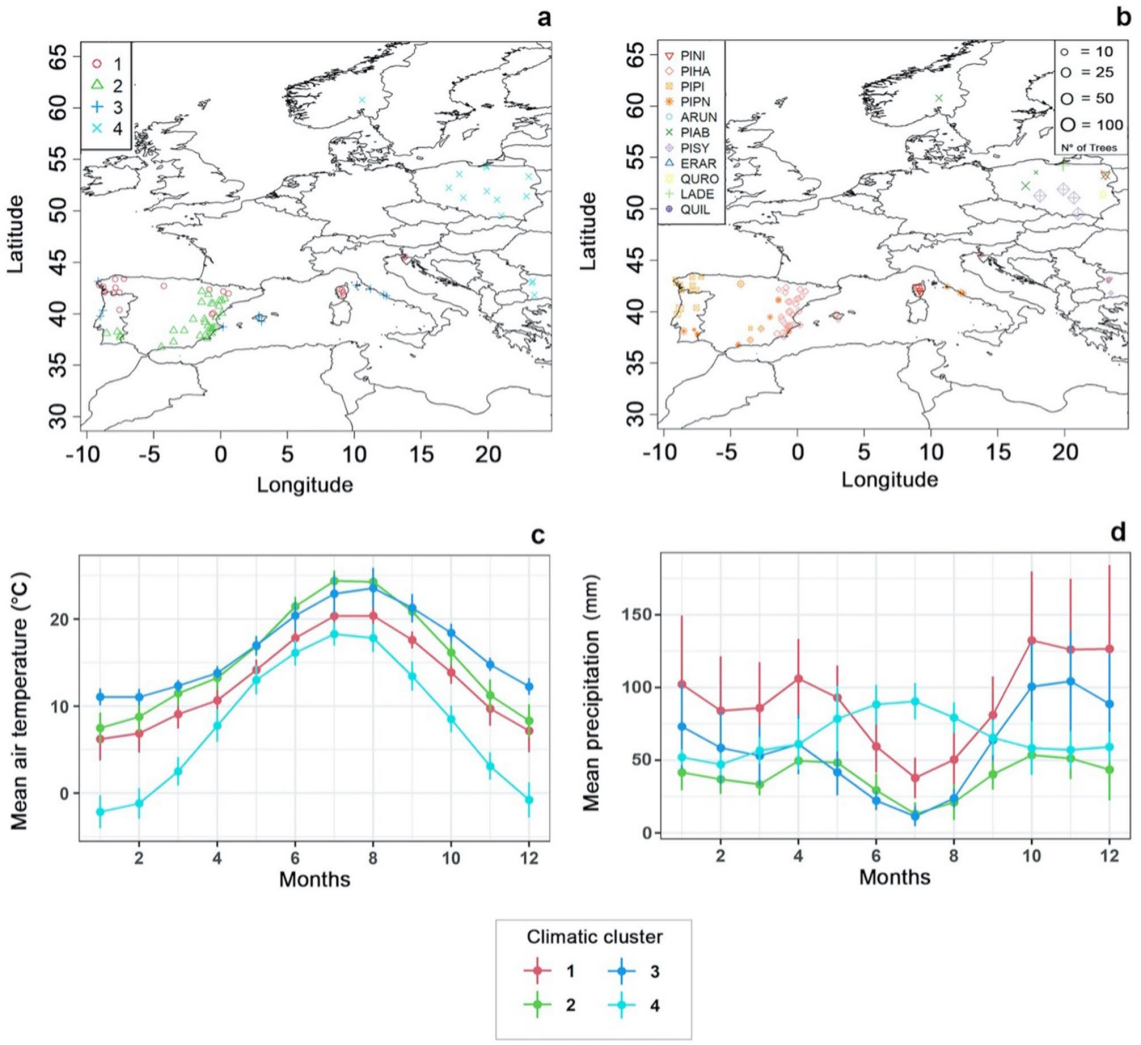
Geographic distribution of study sites and climatic clustering. (**a**) Geographical distribution of the study sites based on the cluster they belong to. (**b**) Represented species and number of trees sampled at each site. **c** Mean air temperature for each climatic cluster. (**d**) Mean precipitation for each climatic cluster. Cluster 1 includes PINI, PIHA, PIPI; cluster 2 includes PIHA, PIPN, PIPI; cluster 3 includes ARUN, PIPN, ERAR, PIPI, PIHA, QUIL; cluster 4 includes PIAB, PISY, QURO, LADE, PINI. Species abbreviations as in Fig. 1. Maps a, b were created with R package version 5.0.0 (https://CRAN.R-project.org/package=prevR) by Jerzy Piotr Kabala.

The maps in Fig. 5a,b show the geographical distribution of the sites based on their respective clusters and on their sample sizes, respectively. The mean climate conditions (air temperature and precipitation) of the clusters are represented in Fig. 5c,d respectively.

Cluster 1 contains Corsica, the north-western Iberian Peninsula, and Slovenia, and represents climates with higher humidity and lower mean temperatures, especially during summer. Cluster 2 includes most of the sites on the Iberian Peninsula and is characterized by lower precipitation and higher temperatures in comparison to cluster 1. Cluster 3 includes sites characterized by summer temperatures and precipitation sums that are comparable to those of cluster 2, but where winters are warmer and wetter. The north-eastern sites (Poland, Bulgaria, and Norway) belong to cluster 4, which differs strongly from the other clusters in climatic conditions. Cluster 4 sites experience maximum precipitation during summer, and winter temperatures that can drop below zero.

**Figure 6:**
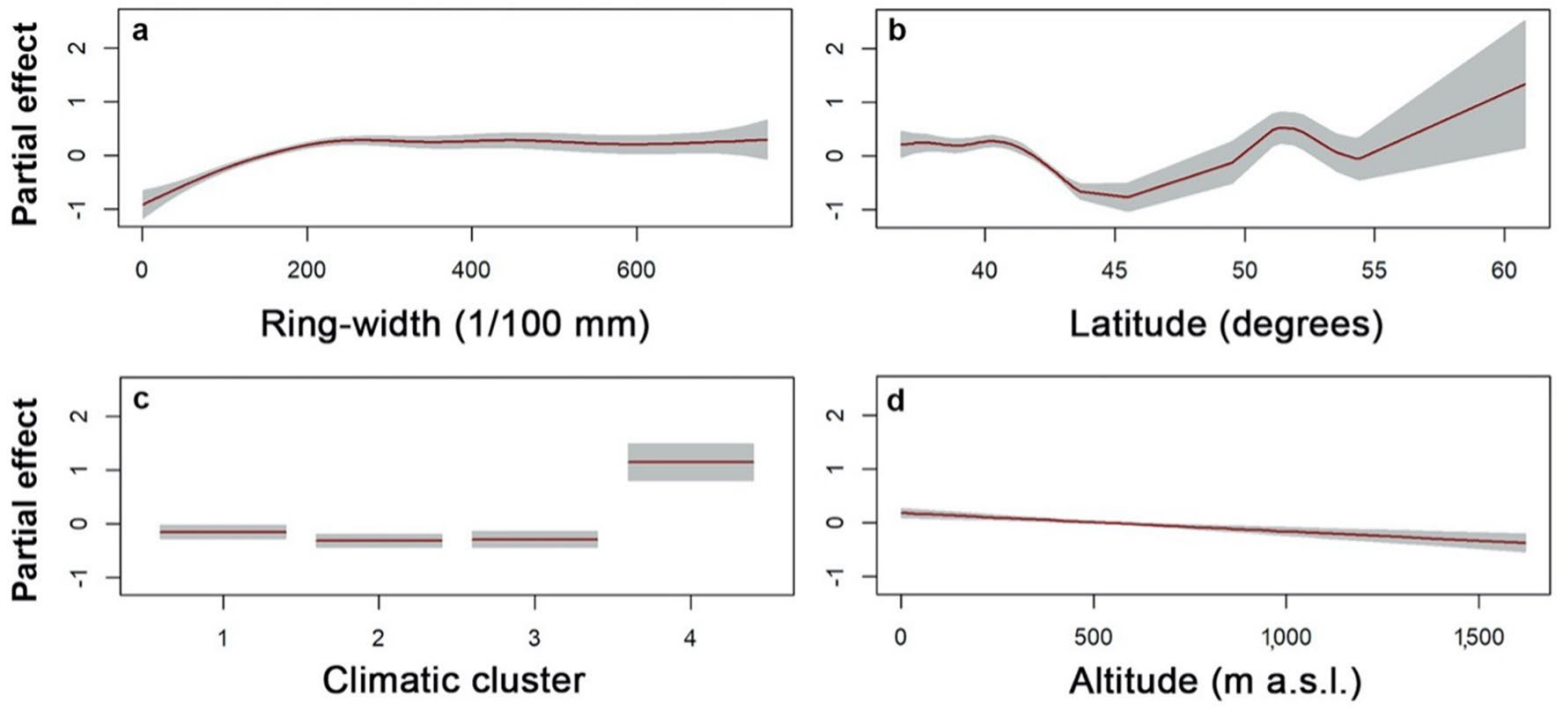
Plots of the modeled effects on IADFs frequency. (**a**) Ring width. (**b**) Latitude. (**c**) Climatic cluster. (**d**) Altitude. The site altitude is included as a linear term, while ring width and latitude are modelled with p-splines, thus accounting for potential non-linearity in their effects; climatic cluster is included as a fixed effect.

A GAM model was fitted to the data to assess the effects of the variables on the IADFs formation (Fig. 6). In the model, the site altitude was included as a linear term, climatic cluster as a fixed effect, while ring width and latitude were modelled with p-splines, thus accounting for potential non-linearity in their effects. The model highlights that the frequency of IADFs increases non-linearly with ring width, reaching a plateau after exceeding a certain ring width (Fig. 6a). There is no clear latitudinal pattern (Fig. 6b), while the climatic cluster 4 is significantly different from the other clusters (Fig. 6c). Finally, IADFs frequency also decreases with altitude (Fig. 6d). Table 1 reports the effects of multiple variables on each species; model coefficients are provided in Supplementary Table 2 and Supplementary Fig. 2. Temperature affects the frequency of IADFs in almost all species, all the coefficients estimated are significant and positive (*p* < 0.05). While for *P. sylvestris*, *Q. ilex, L. decidua* and *Q. robur* correlations between IADFs frequency and temperature are not significant. The coefficients estimated for total annual precipitation are significant (*p* < 0.05) for *A. unedo* and *P. abies*, for which they are negative, and for *Q. ilex*, *P. pinea,* and *P. pinaster*, for which they are positive. For these last three species, a significant positive effect of SPEI has also been detected.

**Table 1:** Effects of climatic variables on IADFs frequency.

Species	Abbreviation	Total annual precipitation	Mean annual temperature	SPEI
*A. unedo*	ARUN	−	+	n.s
*E. arborea*	ERAR	n.s	+	n.s
*L. decidua*	LADE	n.s	n.s	n.s
*P. abies*	PIAB	−	+	n.s
*P. halepensis*	PIHA	n.s	+	n.s
*P. nigra*	PINI	n.s	+	n.s
*P. pinaster*	PIPI	+	+	+
*P. pinea*	PIPN	+	+	+
*P. sylvestris*	PISY	n.s	n.s	n.s
*Q. ilex*	QUIL	+	n.s	+
*Q. robur*	QURO	n.s	n.s	n.s

+ = positive effect, −  = negative effect, n.s. = not significant. Significance level α < 0.05. All the model coefficients are reported in Supplementary Table 2.

## Discussion

This study is the first to analyze IADFs frequency across a broad network of sites representing a large range of climatic conditions and diverse woody species in Europe. A previous study^29^ analyzed IADFs frequency in three *Pinus* species across Italy and the Iberian Peninsula. The present network includes 89 sites and 11 different species, and it is climatologically representative (in terms of mean annual precipitation and temperature) of 4 cluster groups spanning from semi-arid to temperate. The network over-represents the Mediterranean area, where the occurrence of IADFs has been studied much more intensively, and under-represents the northernmost areas of Europe, where IADF studies are less common^17^. We took this over- and under-representation into account by applying weight-adjusted analyses in the GAM model and the interpretation of the results. Further, the limited number of angiosperm samples has been considered in the model with species-specific coefficients regarding temperature, precipitation and SPEI, thus our findings are not dependent on the limited number of samples for angiosperm species. Our findings confirm those of previous studies: for all studied species, the predisposition to form IADFs depends on tree age^29,30^ and tree-ring width^17,21,31^. In particular, all analyzed species show relatively high IADFs frequencies (> 50%, Fig. 2) in wider rings, which has also been reported in studies on pine species^11,29,32–35^. Wide rings are often associated with favorable conditions for tree growth and a longer growing season^20,24,29,36^. In wider rings, more cells are under differentiation for a longer period, which makes the presence of IADFs more likely as long as the triggering factors occur^31,37^.

The uneven spatial distribution of the species in our IADFs network must be taken into account when looking at spatial patterns in IADFs frequency. Nevertheless, the results clearly indicate that some species, such as *P. abies*, *L. decidua,* and *P. halepensis,* are more prone to IADFs formation than others. High IADFs frequencies have already been reported in gymnosperms growing in temperate climates (e.g., *L. decidua* and *P. abies* in Poland^38,39^), where IADFs occurrence is linked to weather fluctuations occurring during the second part of the growing season^40,41^. IADFs in these species may contribute to safer water transport during climate fluctuations^42^, allowing those species to be more plastic and responsive to climate^39^. On the other hand, *P. halepensis* is the most widely distributed Mediterranean pine species and is well-adapted to growing under xeric conditions^43^. Several studies have found that *P. halepensis* experiences a summer reduction in tree growth followed by a reactivation of cambial activity during rainy autumns, which often leads to the formation of IADFs^16,27,29,35,44–46^. Although the occurrence of IADFs is not related to one single environmental factor, since their formation can be influenced by altitude, aspect, tree species, tree state, tree age, and total ring width^20,21,29,34,38,47^, our network analysis indicates that the formation of IADFs in the majority of species depends primarily on warm growing season temperatures (Fig. 5, Table 1). High temperatures such as those recorded in the last decades can induce stomata closure at all canopy levels, especially when coupled with intense drought events^48^. Such events might also force trees to adapt their phenology and start growing earlier in spring^49,50^. In those conditions, trees can anticipate growth onset to take advantage of favorable conditions (i.e., day length, temperature) and maximize growth rates before the start of summer drought^51^. However, anticipating the onset of the growing season has an associated risk: high temperatures during spring and summer drought events^6^. Thus, the longer growing seasons observed at several Mediterranean sites are associated with two main growth peaks interrupted by the drought-induced cessation of summer growth^44^. This bimodal growth pattern is also associated with the formation of IADFs and has been reported in several conifer species (*P. halepensis*^16,44,45,52,53^; *P. pinaster*^33,40,54^; *J. thurifera*^46,55^; *P. pinea*^11^) and in several hardwood species (*Q. ilex*^56^; *A. unedo*^25,57^). In *A. unedo*, an evergreen broadleaf, and *P. abies*, an evergreen conifer, total annual precipitation seems to play an important role in IADF formation. Indeed, we found a higher frequency of IADFs when precipitation is low. Evergreen broadleaved trees grow primarily in the very dry and warm areas of the Mediterranean and suffer from water shortage during summer^6,25^. *A. unedo* in particular strongly reduces its hydraulic conductivity and photosynthetic activity during dry years, and invests stored carbohydrates in structures that guarantee tree survival^6^. Thus, IADFs may be produced to guarantee sufficient mechanical strength against cavitation, and hydraulic safety by modifying tracheid dimensions and pit morphology. On the other hand, *P. abies* is highly dependent on total precipitation^58^; the formation of IADFs in the earlywood of this species is determined by drought conditions^39^. The role of precipitation in IADFs formation is also relevant for *Q. ilex*, *P. pinea,* and *P. pinaster*. Rainfall during the growing season and soon after a cambial stop triggered by drought could favor the resumption of cambial activity leading to the formation of IADFs in latewood^11,29,32^. For instance, in Mediterranean coastal areas, *Q. ilex* can resume growth in response to autumn precipitation, resulting in an “extra” growth band in the annual ring^23^. All of those species are drought tolerant and able to benefit from sporadic rain events during drought^59^. The significant positive correlations between IADFs formation of the three aforementioned species and the multiscale drought index underpins the importance of precipitation for IADF formation^60^. In addition, increased water availability can support higher cell production rates and thereby induce the formation of wider tree rings and potentially containing IADFs.

Our study presents broad-scale patterns of relationships between IADFs and climate data across sites and different tree species over a wide climatic gradient in Europe. At this scale, clustered distribution patterns seem to be larger and more important than local climate variations. The two functional groups (gymnosperms and angiosperms) show similar overall responses in terms of reaction to climate-related triggering factors, although these are based on different seasonal and physiological mechanisms. In particular, IADFs are more frequent at the southern coastal Mediterranean sites, where growing seasons are generally longer. Indeed, high temperature is the common factor influencing the majority of sites and species. To the best of our knowledge, this is the first study to show that IADFs can be used as proxies for past air temperatures with intra-annual resolution. Species with bimodal growth patterns are able to reactivate cambial activity and tolerate drought during their growth. It should be noted that our samples are collected predominantly in the Mediterranean area. It is therefore possible that some of the relationships presented here will differ in strength and direction if more samples from temperate and continental climates areas are included. Expanding this study with more data (especially from other regions) will help to understand possible differences between species and sites. It could also help to better identify species that will respond positively to climate change and the impact that increasing temperatures will have on forest ecosystems.

## Methods

### Site and IADFs European network

In this study, we established a broad tree-ring network from published and unpublished studies covering an area extending from 35°N to 62°N and 10°W to 25°E. This dataset includes data previously used for local and regional studies. A total of 89 different sites from eight European countries and representing eleven different tree species were analyzed (Supplementary Table 1).

### IADFs identification

Great efforts have been put into developing a standardized classification of the different types of IADFs. The approach that classifies IADFs according to their relative position within the tree ring has mostly been used in gymnosperms and has proven to be very consistent^11,20^. In this study, we examined 11 different species with diverse wood anatomical characteristics. Considering this variability in wood anatomy, we decided to maintain a conservative criterion, taking into account only the presence or absence of IADFs within the rings, regardless of their position. All participating research groups identified IADFs following a standardized method^61^.

### IADFs frequencies

IADFs frequencies can be calculated as a simple proportion of the tree rings showing an IADF in a specific year, although other studies^31,62^ have demonstrated how to remove the possible effects of variables such as sample size, age trend, and ring width to improve the climatic signal^63^. Given the variability in the species and geographical locations examined in this study, we standardized our IADFs frequency chronologies using four different methods. We were thus able to test the methodologies individually and find the strongest correlations between IADFs frequency proportions and climate records (Supplementary Fig. 1). The simple relative frequency of IADFs per year (F) was calculated as a ratio:

1 $$F=\frac{N}{n}$$

where ***N*** is the number of cores showing an IADF in a given year, and ***n*** is the total number of cores in that year. To take into account the change in sample depth over time, a stabilized IADFs frequency, ƒ, was calculated as:

2 $${\text{f}} = {\text{F }} \times { }\surd n$$

where F is the relative frequency of IADFs^63^. We calculated two additional IADFs frequencies, the first detrending by age and using a 3-parameter Weibull function (Supplementary Fig. 1a,b) the second detrending by tree-ring width using a 3-parameter Chapman function^31^ (Supplementary Fig. 1c).

### Climate correlations

Total precipitation and monthly mean, maximum, and minimum air temperature, taken from the ERA5 dataset^64^ were correlated for the common period 1979–2000 with IADFs frequency using the Pearson coefficient (*p* < 0.05). Climatic data for each site were extracted from the dataset in Google Earth Engine^65^. In addition, we used the Standardized Precipitation Evapotranspiration Index as an indicator of drought^60,66^. Data were retrieved from the Global SPEI database (https://spei.csic.es; reference period 1979–2016).

We performed a cluster analysis to group sites with similar climates using the PAM (Partitioning Around Medoids) algorithm^67^ which is the extension of k-means clustering algorithm, implemented in the R package cluster^68^. The variables used for computing the distance matrix were the monthly mean, minimum, and maximum temperature for each month, total precipitation, and site coordinates. All variables were standardized before performing the clustering procedure, and the Euclidean distance was used as the distance metric. To select the optimal number of clusters, a silhouette method was applied which minimize the distance between points in a cluster. The number of groups with the highest silhouette value was chosen. The optimal number of groups was four; accordingly, sites were clustered in four climatic groups. The mean annual air temperature and total annual precipitation were calculated from the dataset for each site and for each year. The annual mean SPEI (SPEI calculated at a time scale of 6 months^66^) for each site and each year was obtained from the gridded SPEI data. The mean ring width for each year, sampling site, and species was calculated. A Generalize Additive Model (GAM) was then fitted to the data using the *gamlss* R package^69^. The variables included in the model were: altitude, latitude, mean air temperature, total annual precipitation, mean ring width, mean annual SPEI, species, and climatic cluster. In addition, the four clusters from the cluster analysis were included in the model to assess the effects of different intra-annual patterns of precipitation and temperature on the process of IADFs formation. The normal distribution of the model residuals was assessed with the Cramer-Von-Mises test, implemented in the nortest R package^70^.

The GAM model is as follows:

3 $${IADF}_{cf}=alt+f\left(lat\right)+f\left(rw\right)+tp*species+temp*species+SPEI*species+cluster$$

where: alt = site altitude (meters above sea level); lat = site latitude (degrees); rw = mean ring width (1/100 mm); species = species; tp = total annual precipitation; temp = mean annual temperature; SPEI = mean annual SPEI: annual mean of the monthly SPEI values; cluster = climatic cluster according to the cluster analysis.

A p-splines estimation method was used to represent the non-linear relationship between ring width, latitude, and IADFs frequency. The interaction terms “species*precipitation”, “species*temperature” and “species*SPEI” allowed us to evaluate species-specific responses to those environmental variables. We chose the Zero Adjusted Gamma distribution as the family distribution because it considers the massive presence of zeroes in the response variable, which corresponds to the absence of IADFs. The R^2^ of the model is 0.50, and the residuals do not deviate significantly from normality (*p* > 0.05, Cramer-Von Mises test).

## Supplementary Information

Supplementary Information.

## Data Availability

The data presented in this study are available on request from the corresponding author.
