# Supplementary material for: Intra-annual density fluctuations in tree rings are proxies of air temperature across Europe
Source: Sci Rep. 2023 Jul 29;13:12294. doi: 10.1038/s41598-023-39610-8 (PMC10387074; doi:10.1038/s41598-023-39610-8)
Supplement: Supplementary file 1 — Supplementary Information. [file 41598_2023_39610_MOESM1_ESM.pdf]

Intra-annual density fluctuations in tree rings are proxies of air temperature across Europe

Battipaglia, G.[1*], Kabala J.P.[1], Pacheco-Solana A.[1,2], Niccoli F.[1], Bräuning, A.[3], Campelo, F.[4], Cufar, K.[5], de Luis, M.[6], De Micco, V.[7], Klisz, M.[8], Koprowski, M.[9], Garcia-Gonzalez, I.[10], Nabais, C.[4], Vieira, J.[4,11], Wrzesiński, P.[8], Zafirov, N.[12], Cherubini, P.[13,14]

Supplementary Fig. 1: Stabilized IADF frequency chronologies using 4 different detrending methods.

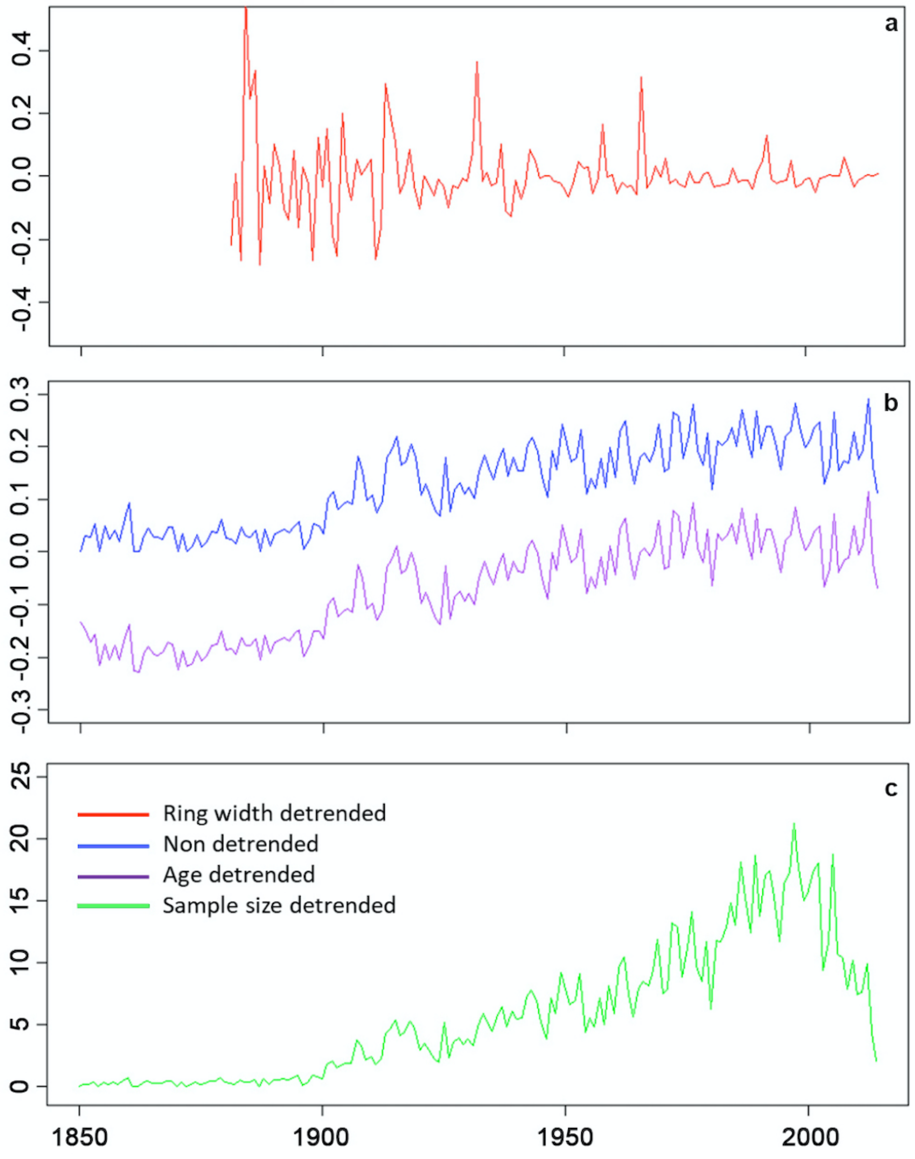

Ring width detrended where a minimum number of five trees was available.

Supplementary Fig. 2: ***Observed vs fitted values according to the GAM model.***

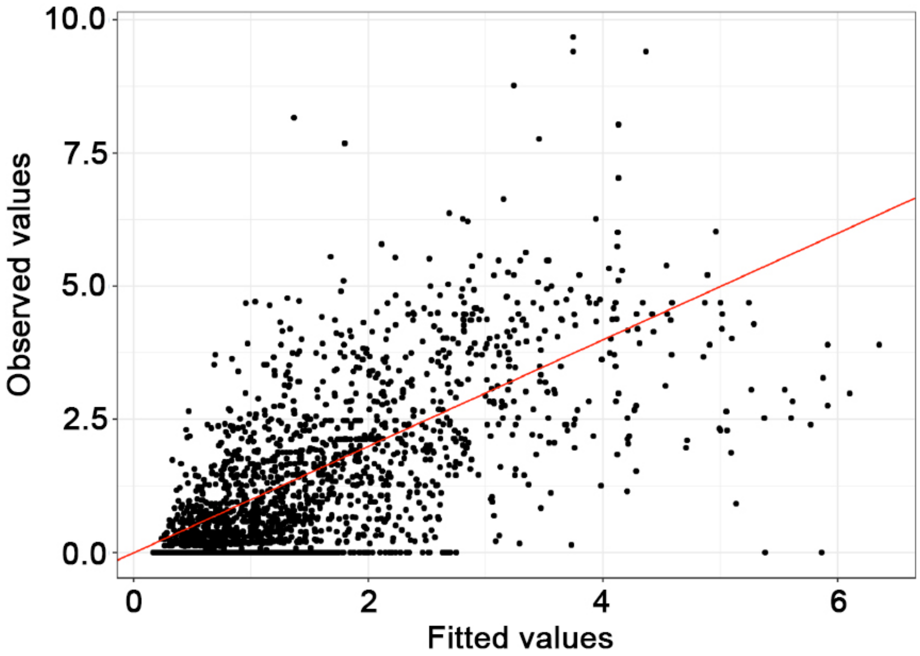

Supplementary Table 1: Details of each analyzed site in the network and the relevant references.

Country	Species	Abbreviation	Site code	Lat	Lon	Alt (m a.s.l.)	N° of samples	N° of rings	Start year	End year	Reference
**BULGARIA**	Pinus nigra	PINI	SVB	43.14	23.35	975	24	839	1979	2013	**Zafirov et al., unpublished**
	Pinus sylvestris	PISY	PRN	41.81	23.47	1150	9	315	1979	2013	
	Pinus sylvestris	PISY	SVB	43.14	23.35	975	24	839	1979	2013	
	Pinus nigra	PINI	SVG	42.99	23.28	650	15	540	1979	2014	
	Pinus sylvestris	PISY	SVG	42.99	23.28	650	15	540	1979	2014	
**FRANCE**	Pinus nigra	PINI	CB	41.78	9.24	1300	24	681	1979	2007	**Szymczak et al., 2014**
	Pinus nigra	PINI	AC	42.40	8.92	1600	66	1870	1979	2009	
	Pinus nigra	PINI	AS	41.83	9.22	1375	42	1270	1979	2009	
	Pinus nigra	PINI	CN	42.06	9.16	1500	61	1799	1979	2009	
	Pinus nigra	PINI	CP	42.08	9.16	1600	26	764	1979	2009	
	Pinus nigra	PINI	PI	42.32	9.08	1620	52	1525	1979	2009	
**ITALY**	Pinus pinea	PIPN	CPPN	41.74	12.40	10	21	513	1979	2003	**Zalloni et al., 2016**
	Pinus pinea	PIPN	DFPN	42.44	11.22	10	14	349	1979	2003	
	Pinus pinea	PIPN	FRPN	41.86	12.20	10	22	737	1979	2012	
	Arbutus unedo	ARUN	AUFM	42.77	10.20	420	10	280	1979	2006	**Battipaglia et al., 2010**
	Arbutus unedo	ARUN	AUTP	42.77	10.18	460	9	106	1979	2006	
	Erica arborea	ERAR	EAFM	42.77	10.20	420	8	224	1979	2006	**Battipaglia et al., 2014**
	Quercus ilex	QUIL	QITP	42.77	10.18	460	9	110	1979	2006	**Zalloni et al., 2016; 2018**
	Quercus ilex	QUIL	QUIFM	42.77	10.20	420	8	188	1979	2006	
	Pinus pinaster	PIPI	PPFM	42.77	10.20	420	8	232	1979	2007	
	Pinus pinaster	PIPI	PPTP	42.77	10.18	460	9	257	1979	2007	
**NORWAY**	Picea abies	PIAB	BJU	60.78	10.60	350	48	851	1979	2002	**Steffenrem, unpublished**
**POLAND**	Larix decidua	LADE	MLY	54.22	19.91	60	314	4988	1979	2006	**Klisz et al., 2016; 2019**
	Larix decidua	LADE	ZAP	54.41	19.85	9	330	4938	1979	2006	
	Picea abies	PIAB	KN	53.33	23.06	155	199	6449	1979	2014	
	Picea abies	PIAB	KR	52.24	17.08	80	138	4692	1979	2014	
	Picea abies	PIAB	TUSW	53.55	17.86	143	11	292	1979	2005	

	Quercus robur	QURO	LUDA	51.41	22.89	158	18	558	1979	2009	**Koprowski et al., unpublished data**
	Pinus sylvestris	PISY	BL	51.07	20.69	311	225	8588	1979	2016	**Wrzesinski & Klisz unpublished data**
	Pinus sylvestris	PISY	LI	51.90	19.91	195	225	8550	1979	2016	
	Pinus sylvestris	PISY	PO	49.50	21.00	653	225	8545	1979	2016	
	Pinus sylvestris	PISY	SI	51.26	18.15	166	225	8436	1979	2016	
**PORTUGAL**	Pinus pinea	PIPN	BEME	37.71	-7.49	190	14	336	1979	2002	**Campelo et al. 2007; 2013, Nabais et al. 2014; Vieira et al. 2009; 2010; Zalloni et al., 2016**
	Pinus pinea	PIPN	BESE	37.90	-7.50	210	10	240	1979	2002	
	Pinus pinea	PIPN	EVPO	38.25	-7.73	270	10	240	1979	2002	
	Pinus pinea	PIPN	ASPI	38.10	-8.52	160	50	1250	1979	2003	
	Pinus pinaster	PIPI	LEMG	39.76	-8.99	33	80	2222	1979	2006	
	Pinus pinaster	PIPI	SG	42.00	-8.00	1500	36	1032	1979	2007	
	Pinus pinaster	PIPI	TCHA	40.33	-8.80	16	120	3597	1979	2008	
	Pinus pinaster	PIPI	SEPP	40.38	-7.55	1050	56	1786	1979	2010	
**SLOVENIA**	Pinus halepensis	PIHA	DKN	45.55	13.81	109	19	469	1979	2003	**Novak et al., 2013**
	Pinus halepensis	PIHA	KRK	45.46	13.69	136	30	750	1979	2003	
**SPAIN**	Pinus halepensis	PIHA	ALC	38.68	-0.36	674	30	654	1979	2000	**Novak et al., 2013; Zalloni et al., 2016**
	Pinus halepensis	PIHA	BIA	38.62	-0.77	806	29	633	1979	2000	
	Pinus halepensis	PIHA	CRE	38.29	-0.77	285	41	895	1979	2000	
	Pinus halepensis	PIHA	JAV	38.73	0.19	96	60	1302	1979	2000	
	Pinus halepensis	PIHA	MON	40.06	-0.54	569	31	703	1979	2001	
	Pinus halepensis	PIHA	ZOR	40.74	-0.11	857	27	609	1979	2001	
	Pinus halepensis	PIHA	CHI	41.24	-0.09	160	10	250	1979	2003	
	Pinus halepensis	PIHA	EBA	42.17	-1.40	365	9	202	1979	2003	

	Pinus halepensis	PIHA	GUA	38.10	-0.65	15	41	1299	1979	2003	
	Pinus halepensis	PIHA	ORO	40.06	0.12	1	30	750	1979	2003	
	Pinus halepensis	PIHA	REQ	39.47	-1.20	721	30	750	1979	2003	
	Pinus halepensis	PIHA	JAL	39.19	-1.15	571	47	1145	1979	2004	
	Pinus halepensis	PIHA	FNT	38.67	-0.54	1022	24	648	1979	2005	
	Pinus halepensis	PIHA	ALL	40.98	-0.57	595	31	868	1979	2006	
	Pinus halepensis	PIHA	AYE	42.32	-0.84	924	33	917	1979	2006	
	Pinus halepensis	PIHA	CAP	41.75	-0.50	738	58	1588	1979	2006	
	Pinus halepensis	PIHA	CAS	41.29	0.07	166	28	780	1979	2006	
	Pinus halepensis	PIHA	EST	41.97	0.61	502	26	728	1979	2006	
	Pinus halepensis	PIHA	FRA	41.47	0.32	340	27	756	1979	2006	
	Pinus halepensis	PIHA	GIL	39.67	-0.35	175	26	728	1979	2006	
	Pinus halepensis	PIHA	GRA	42.16	0.20	168	30	839	1979	2006	
	Pinus halepensis	PIHA	OLI	40.99	-0.69	530	27	756	1979	2006	
	Pinus halepensis	PIHA	VLL	41.88	-0.91	452	27	756	1979	2006	
	Pinus halepensis	PIHA	CAT	37.61	-1.01	116	27	783	1979	2007	
	Pinus halepensis	PIHA	FUE	37.94	-1.12	138	26	754	1979	2007	
	Pinus halepensis	PIHA	SES	37.86	-1.52	846	29	841	1979	2007	
	Pinus pinea	PIPN	DAR	41.14	-1.41	937	31	1346	1979	2007	

	Pinus halepensis	PIHA	MAN	38.52	-0.64	845	65	1760	1979	2009	
	Pinus halepensis	PIHA	MAS	38.50	-0.60	762	39	1198	1979	2009	
	Pinus halepensis	PIHA	FHI	38.83	-0.93	680	28	924	1979	2011	
	Pinus halepensis	PIHA	GI2	39.67	-0.31	140	23	754	1979	2011	
	Pinus halepensis	PIHA	PA1	39.69	2.86	120	26	849	1979	2011	
	Pinus halepensis	PIHA	PA2	39.79	2.89	386	25	742	1979	2011	
	Pinus halepensis	PIHA	PA3	39.27	3.05	14	22	696	1979	2011	
	Pinus halepensis	PIHA	RAG	39.97	-0.65	959	29	957	1979	2011	
	Pinus halepensis	PIHA	DAR	41.14	-1.41	937	31	1346	1979	2007	**Zalloni et al., 2016**
	Pinus halepensis	PIHA	MDM	36.76	-4.42	135	26	1454	1979	2012	
	Pinus halepensis	PIHA	PSC	38.36	-2.72	1030	29	1627	1979	2012	
	Pinus halepensis	PIHA	SDH	37.27	-3.54	1120	29	1695	1979	2012	
	Pinus pinaster	PIPI	DES	38.37	-3.51	600	20	676	1979	2012	
	Pinus pinaster	PIPI	PSC	38.36	-2.72	1050	29	1627	1979	2012	
	Pinus pinaster	PIPI	SDH	37.25	-3.49	1300	29	1695	1979	2012	
	Pinus pinea	PIPN	MDM	36.76	-4.42	135	26	1454	1979	2012	
	Pinus pinea	PIPN	RBA	39.48	-2.05	800	23	782	1979	2012	
	Pinus pinea	PIPN	GUA	38.10	-0.65	15	41	1299	1979	2003	
	Pinus pinaster	PIPI	COR	42.62	-8.78	20	22	570	1979	2004	**Zalloni et al., 2016; Rozas et al., 2011**
	Pinus pinaster	PIPI	ALO	42.08	-8.68	530	21	571	1979	2006	
	Pinus pinaster	PIPI	BAR	42.53	-7.85	620	23	644	1979	2006	

	Pinus pinaster	PIPI	CAP	43.67	-7.98	340	58	1588	1979	2006	
	Pinus pinaster	PIPI	INS	43.13	-9.15	10	14	379	1979	2006	
	Pinus pinaster	PIPI	MCU	43.37	-7.88	590	19	532	1979	2006	
	Pinus pinaster	PIPI	MUR	42.80	-9.07	155	21	587	1979	2006	
	Pinus pinaster	PIPI	TRA	43.42	-7.22	640	23	644	1979	2006	
	Pinus pinaster	PIPI	VER	42.07	-7.55	855	23	644	1979	2006	
	Pinus pinaster	PIPI	VIG	42.20	-8.65	365	19	532	1979	2006	**Zalloni et al., 2016**

Supplementary Table 2: Coefficients estimated for the GAM model.

	Estimate	Std. Error	t value	Pr(>|t|)	Significance
**(Intercept)**	-3.93	1.16	-3.4	6.83E-04	***
**pb(width)**	1.23E-03	1.40E-04	8.762	< 2e-16	***
**pb(LAT)**	0.06	0.02	2.923	3.49E-03	**
**clim.group2**	0.20	0.10	2.059	0.04	*
**clim.group3**	0.36	0.13	2.787	0.01	**
**clim.group4**	-0.32	0.11	-2.98	2.91E-03	**
**clim.group5**	0.09	0.17	0.512	0.61	
**clim.group6**	1.30	0.28	4.582	4.82E-06	***
**ALT**	-6.34E-04	8.01E-05	-7.918	3.56E-15	***
**mean_annual_temperature:SPECIESARUN**	0.21	0.06	3.711	2.11E-04	***
**mean_annual_temperature:SPECIESERAR**	0.14	0.07	2.075	0.04	*
**mean_annual_temperature:SPECIESLADE**	0.04	0.17	0.253	0.80	
**mean_annual_temperature:SPECIESPIAB**	0.20	0.05	4.123	3.86E-05	***
**mean_annual_temperature:SPECIESPIHA**	0.07	0.03	2.356	0.02	*
**mean_annual_temperature:SPECIESPINI**	0.16	0.04	3.81	1.42E-04	***
**mean_annual_temperature:SPECIESPIPI**	0.09	0.03	3.239	1.22E-03	**
**mean_annual_temperature:SPECIESPIPN**	0.07	0.03	2.505	0.01	*
**mean_annual_temperature:SPECIESPISY**	0.15	0.11	1.294	0.20	
**mean_annual_temperature:SPECIESQUIL**	0.14	0.07	1.94	0.05	.
**mean_annual_temperature:SPECIESQURO**	0.13	0.13	0.979	0.33	
**SPECIESARUN:total_precipitation**	-2.83	1.26	-2.248	0.02	*
**SPECIESERAR:total_precipitation**	0.46	1.56	0.296	0.77	
**SPECIESLADE:total_precipitation**	-1.47	1.64	-0.892	0.37	
**SPECIESPIAB:total_precipitation**	-3.91	0.66	-5.91	0.00	***
**SPECIESPIHA:total_precipitation**	0.29	0.18	1.646	0.10	.
**SPECIESPINI:total_precipitation**	-0.64	0.55	-1.147	0.25	
**SPECIESPIPI:total_precipitation**	1.15	0.10	11.922	< 2e-16	***
**SPECIESPIPN:total_precipitation**	1.10	0.26	4.301	1.76E-05	***

**SPECIESPISY:total_precipitation**	-1.49	1.23	-1.211	0.23	
**SPECIESQUIL:total_precipitation**	0.95	1.81	0.523	0.03	*
**SPECIESQURO:total_precipitation**	-3.27	1.49	-2.196	0.63	
**SPECIESARUN:SPEI06_MEAN**	0.54	0.34	1.568	0.12	
**SPECIESERAR:SPEI06_MEAN**	-0.06	0.37	-0.171	0.86	
**SPECIESLADE:SPEI06_MEAN**	-0.03	0.26	-0.122	0.90	
**SPECIESPIAB:SPEI06_MEAN**	0.06	0.17	0.329	0.74	
**SPECIESPIHA:SPEI06_MEAN**	0.11	0.06	1.857	0.06	.
**SPECIESPINI:SPEI06_MEAN**	0.13	0.15	0.857	0.39	
**SPECIESPIPI:SPEI06_MEAN**	0.24	0.05	4.72	2.48E-06	***
**SPECIESPIPN:SPEI06_MEAN**	0.08	0.09	0.944	0.02	*
**SPECIESPISY:SPEI06_MEAN**	-0.02	0.33	-0.06	0.95	
**SPECIESQUIL:SPEI06_MEAN**	0.16	0.41	0.388	0.02	*
**SPECIESQURO:SPEI06_MEAN**	0.10	0.30	0.342	0.73	

Battipaglia, G. *et al.* Variations of vessel diameter and δ13C in false rings of Arbutus unedo L. reflect different environmental conditions. *New Phytol.* **184**, 1099–1112 (2010).

Battipaglia, G. *et al.* Drought impact on water use efficiency and intra-annual density fluctuations in Erica arborea on Elba (Italy). *Plant, Cell Environ.* **37**, 382–391 (2014).

Campelo, F., Nabais, C., Freitas, H. & Gutiérrez, E. Climatic significance of tree-ring width and intra-annual density fluctuations in Pinus pinea from a dry Mediterranean area in Portugal. *Ann. For. Sci.* (2007) doi:10.1051/forest:2006107.

Campelo, F., Vieira, J. & Nabais, C. Tree-ring growth and intra-annual density fluctuations of Pinus pinaster responses to climate: Does size matter? *Trees - Struct. Funct.* (2013) doi:10.1007/s00468-012-0831-3.

Klisz, M. *et al.* Effect of provenance and climate on intra-annual density fluctuations of Norway spruce Picea abies (L.) Karst. in Poland. *Agric. For. Meteorol.* (2019) doi:10.1016/j.agrformet.2019.02.013.

Klisz, M., Koprowski, M., Ukalska, J. & Nabais, C. Does the genotype have a significant effect on the formation of intra-annual density fluctuations? A case study using Larix decidua from Northern Poland. *Front. Plant Sci.* (2016) doi:10.3389/fpls.2016.00691.

Nabais, C., Campelo, F., Vieira, J. & Cherubini, P. Climatic signals of tree-ring width and intra-annual density fluctuations in Pinus pinaster and Pinus pinea along a latitudinal gradient in Portugal. *Forestry* (2014) doi:10.1093/forestry/cpu021.

Novak, K., Čufar, K., De Luis, M., Sánchez, M. A. S. & Raventós, J. Age, climate and intra-annual density fluctuations in Pinus halepensis in Spain. *IAWA J.* **34**, 459–474 (2013).

Rozas, V., García-González, I. & Zas, R. Climatic control of intra-annual wood density fluctuations of Pinus pinaster in NW Spain. *Trees - Struct. Funct.* (2011) doi:10.1007/s00468-010-0519-5.

Szymczak, S. *et al.* Combining wood anatomy and stable isotope variations in a 600-year multi-parameter climate reconstruction from corsican black pine. *Quat. Sci. Rev.* (2014) doi:10.1016/j.quascirev.2014.07.010.

Vieira, J., Campelo, F. & Nabais, C. Age-dependent responses of tree-ring growth and intra-annual density fluctuations of Pinus pinaster to Mediterranean climate. *Trees - Struct. Funct.* (2009) doi:10.1007/s00468-008-0273-0.

Vieira, J., Campelo, F. & Nabais, C. Intra-annual density fluctuations of Pinus pinaster are a record of climatic changes in the western Mediterranean region. *Can. J. For. Res.* (2010) doi:10.1139/X10-096.

Zalloni, E. *et al.* Climatic signals from intra-annual density fluctuation frequency in mediterranean pines at a regional scale. *Front. Plant Sci.* **7**, 579 (2016).

Zalloni, E., Battipaglia, G., Cherubini, P., Saurer, M. & De Micco, V. Contrasting physiological

responses to Mediterranean climate variability are revealed by intra-annual density fluctuations in tree rings of Quercus ilex L. And Pinus pinea L. And. *Tree Physiol.* (2018) doi:10.1093/treephys/tpy061.
